# Ensemble transfer learning for the prediction of anti-cancer drug response

**DOI:** 10.1038/s41598-020-74921-0

**Published:** 2020-10-22

**Authors:** Yitan Zhu, Thomas Brettin, Yvonne A. Evrard, Alexander Partin, Fangfang Xia, Maulik Shukla, Hyunseung Yoo, James H. Doroshow, Rick L. Stevens

**Affiliations:** 1 Computing, Environment and Life Sciences, Argonne National Laboratory, Lemont, IL 60439 USA; 2 Frederick National Laboratory for Cancer Research, Leidos Biomedical Research, Inc., Frederick, MD 21702 USA; 3 Developmental Therapeutics Branch, National Cancer Institute, Bethesda, MD 20892 USA; 4 Department of Computer Science, The University of Chicago, Chicago, IL 60637 USA

**Keywords:** Predictive medicine, Virtual screening, Computer modelling, Machine learning, Virtual drug screening

## Abstract

Transfer learning, which transfers patterns learned on a source dataset to a related target dataset for constructing prediction models, has been shown effective in many applications. In this paper, we investigate whether transfer learning can be used to improve the performance of anti-cancer drug response prediction models. Previous transfer learning studies for drug response prediction focused on building models to predict the response of tumor cells to a specific drug treatment. We target the more challenging task of building general prediction models that can make predictions for both new tumor cells and new drugs. Uniquely, we investigate the power of transfer learning for three drug response prediction applications including drug repurposing, precision oncology, and new drug development, through different data partition schemes in cross-validation. We extend the classic transfer learning framework through ensemble and demonstrate its general utility with three representative prediction algorithms including a gradient boosting model and two deep neural networks. The ensemble transfer learning framework is tested on benchmark in vitro drug screening datasets. The results demonstrate that our framework broadly improves the prediction performance in all three drug response prediction applications with all three prediction algorithms.

## Introduction

Cancer is a complex, dynamic, and heterogenous disease. Patients with the same cancer histology can respond differently to the same anti-cancer therapy^1^. Multiple in vitro drug screening studies have been conducted generating data about drug efficacy on cancer cell lines (CCLs)^2–6^. Due to the heterogeneity of cancer, an accurate prediction of the response of cancer cells to a drug treatment is of paramount importance for therapeutics development and patient care. There are three major applications for drug response prediction including drug repurposing, precision oncology, and new drug development. The goal of drug repurposing is to examine whether an existing drug used to treat a specific cancer indication can be used to treat another cancer indication. In drug repurposing, both the drug and cancer are not new but their combination has not been previously tested. For precision oncology, the goal is to identify an existing drug to treat a new cancer case that has not been investigated or treated before. The development of new drugs requires predicting the response of known cancer cases under the treatment of a new drug that has not been tested before.

Various methods and analysis schemes have been developed and used to predict anti-cancer drug response, which can be categorized in different ways. Conventional machine learning methods, such as ridge and elastic net regressions^7^, random forests^8^, modified rotation forest^9^, and support vector machine^10^, have been used in drug response prediction. Recently, deep learning methods have started to play an increasingly important role^11–15^. Some studies predicted dose-dependent cell growth inhibition^11^, and many others predicted dose-independent drug response measurements, such as the area under the dose response curve (AUC) and the half maximal inhibitory concentration (IC_50_)^10,13,16,17^. Some analyses have constructed a prediction model for an individual cancer type and/or drug^13,18,19^, and others have built general prediction models covering multiple cancer types and/or drugs^11,12,14,16,17^. While transcriptomic data and other omics data, such as genomic and proteomic data, have been used for the prediction of drug response, transcriptomic data have been shown to be the most predictive among all omic modalities^7,8^. Most works have targeted the prediction of single drug response^13,16,17,20^, though some predicted the response of drug combinations^11,21–23^. While many prediction models take tumor and drug molecular features as inputs to predict drug response, methods like Bayesian efficient multiple kernel learning^8,24^, neighbor-based collaborative filtering^25,26^, weighted graph regularized matrix factorization^27^, and kernelized similarity based regularized matrix factorization^28^ have been developed to predict drug response based on similarity measures between tumors and drugs. Ensemble and multi-task learning frameworks have also been developed for drug response prediction^8,9,24,29^.

In this paper, rather than developing a new algorithm for drug response prediction, we propose a transfer learning framework that can improve the prediction performance of existing algorithms by incorporating prediction patterns learned from other related data. The general goal of transfer learning is to build a high-performance learner for a target domain where data availability is limited using prediction patterns learned from a related source domain with abundant data^30,31^. Transfer learning has been successfully used in many areas, such as text classification^32,33^ and image classification^32,34^. An example of source and target domains in transfer learning can be given using image classification, in which classifiers can be first trained based on the abundant natural images and then be refined based on relatively limited medical images for disease diagnosis^35^. Deep transfer learning implements transfer learning with deep neural network (DNN) models^36–38^. One popular deep transfer learning technique is to transfer the front layers of a DNN model trained in the source domain to the target domain and use it as a feature extractor^37,38^. Based on the target domain data, either the parameters of the back layers are refined or the back layers are removed and new layers are added behind the front layers and trained from scratch. The idea behind this approach is that the DNN model forms an iterative and continuous abstraction process and the front layers may generate features informative in both domains^36^. The model refinement on the target domain data updates parameters in the back layers of DNN models, so that the more abstracted features can be adapted to the target prediction task.

In the context of drug response prediction, the target and source domains of transfer learning can be different drug screening studies/datasets^39^. Differences in experimental protocols, assays, or biological models and drugs used in the studies generate variations between these datasets. It has been reported that the same treatment experiments (i.e. pairs of drugs and CCLs) might have quite different response values in different studies^39^. Supplementary Fig. S1 also shows the distribution of drug response varies between drug screening datasets. Thus, different drug screening datasets and their associated drug response prediction tasks can be taken as related but different domains for the application of transfer learning. There exist several works that applied transfer learning related strategies to drug response prediction. Dhruba et al. utilized one drug screening dataset to help the prediction on another drug screening dataset through transfer learning, which either transforms the two datasets into a unified latent space or transforms one dataset to the space of the other dataset through regression mappings^39^. Turki et al. developed approaches to combine an in vitro drug screening dataset with auxiliary data for predicting patient treatment response^40,41^. Borisov et al. predicted the response of a patient to a drug treatment by building a prediction model for the patient using cell lines similar to the patient evaluated by gene expressions of selected drug-related pathways^42^.

We propose an ensemble transfer learning (ETL) framework for anti-cancer drug response prediction. The ETL framework applies the classic transfer learning scheme that trains a prediction model on the source dataset and then refines it on the target dataset, but extends the scheme through ensemble prediction by training and refining multiple models. Compared with the above existing works, our work makes unique contributions. First, while existing works on transfer learning for drug response prediction focus on building prediction models for a specific drug^39–42^, we target the more challenging task of building general prediction models that are not specific to a drug. Different from drug-specific prediction models, general drug response prediction models are trained on data of multiple drugs. Features of both cancer cells and drugs are used as inputs for general prediction models, while drug-specific models usually use only cancer cell features for prediction. Importantly, general drug response prediction models can make predictions for not only new cancer cases but also new drugs. Due to these differences, existing transfer learning methods for building drug-specific prediction models are not directly applicable for building general drug response prediction models. Our study is the first one to propose a transfer learning framework for building general drug response prediction models and to investigate whether transfer learning can improve the prediction performance in such a setting. Second, we test the power of transfer learning for three different drug response prediction applications including drug repurposing, precision oncology, and new drug development, via different data partition and selection schemes in cross-validation, which to our knowledge has not been investigated before.

There are many choices of prediction algorithms for implementing the proposed ETL framework. We select three representative and generic prediction models including LightGBM^43^ (an efficient gradient boosting decision tree algorithm) and two DNN models of different architectures to implement the analysis pipeline. We apply ETL on multiple in vitro drug screening datasets simulating the three different drug response prediction applications. Baseline analysis schemes using the same prediction models but without ETL are also applied for comparison purpose. Based on the analysis results, we compare the prediction performances obtained with and without transfer learning and also compare between transfer learning using different prediction models for each of the drug response prediction applications.

## Methods

### Framework of analysis scenario

Our study involves four public in vitro drug screening datasets, including the Cancer Therapeutics Response Portal v2 (CTRP)^3^, the Genomics of Drug Sensitivity in Cancer (GDSC)^4^, the Cancer Cell Line Encyclopedia (CCLE)^5^, and the Genentech Cell Line Screening Initiative (GCSI)^6^. Based on the drug response data, AUC values are calculated and taken as the drug response measurements to be predicted through regression analysis. RNA-seq data including expression values of 1927 selected genes are used to represent CCLs. Drugs are represented by 1623 molecular descriptors for modeling analysis. See Section 1 in the Supplementary Information for details about the data and how they have been preprocessed for analysis. Supplementary Table S1 gives the numbers of CCLs, drugs, and treatments (pairs of drugs and CCLs) in each dataset. For transfer learning, we use the two large datasets CTRP and GDSC as the source data and use the two small datasets CCLE and GCSI as the target data, which forms four transfer learning tasks denoted by CTRP → CCLE, CTRP → GCSI, GDSC → CCLE, and GDSC → GCSI.

A goal of our study is to investigate whether ensemble transfer learning can improve the prediction of drug response compared to not using transfer learning. For each transfer learning task, the ETL framework first trains prediction models on the source dataset and then refines them on a part of the target dataset. After refinement, the models are applied on the rest of the target dataset to make ensemble predictions. Details of the ETL analysis scheme will be introduced in the next subsection. The prediction performance of ETL is evaluated based on the ensemble predictions and compared to those of baseline schemes that build prediction models based on only the target data without transfer learning. Two baseline schemes are applied, standard cross-validation (SCV) and ensemble cross-validation (ECV). SCV is the conventional cross-validation scheme, with the prediction performance evaluated in each cross-validation trial. ECV modifies the scheme of SCV via embedding ensemble learning. Specifically, in each cross-validation trial, ECV resamples the training set 10 times to train 10 prediction models. All these models are then applied on the testing set to generate ensemble predictions, based on which the prediction performance is evaluated. The analysis schemes of SCV and ECV are explained in details in Section 2 of the Supplementary Information. Supplementary Fig. S2 shows their analysis flowcharts.

**Figure 1:**
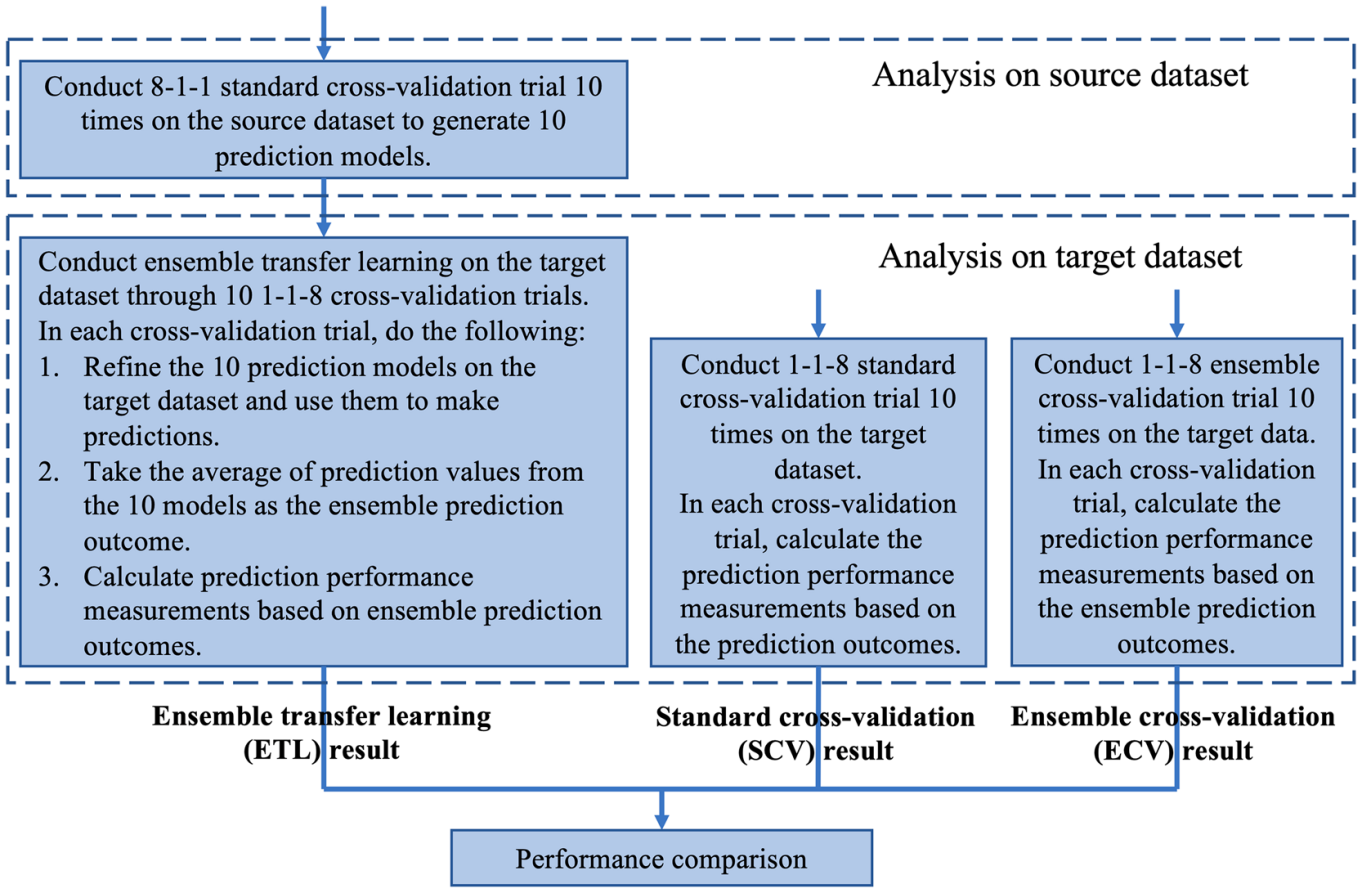
Analysis scenario framework. The analysis scheme on the left is ensemble transfer learning (ETL). The middle and right analysis schemes are standard cross-validation (SCV) and ensemble cross-validation (ECV), respectively, which do not apply transfer learning but instead analyze only the target dataset.

The prediction performances of the three analysis schemes are compared with each other to investigate whether ETL can improve the prediction performance. See Fig. 1 for the framework of the whole analysis scenario. In Fig. 1, 8-1-1 cross-validation means dividing the data into 10 data folds and using 8, 1, and 1 data fold for model training, validation, and testing, respectively. 8-1-1 cross-validation is used at the first step of transfer learning to train models on the source dataset. 1-1-8 cross-validation means dividing the data into 10 data folds and using 1, 1, and 8 data folds for model training/refinement, validation, and testing, respectively. 1-1-8 cross-validation is used for all analyses on the target data, including SCV, ECV, and the second step of transfer learning, to simulate a situation where the training data at the target domain are quite limited. The validation set is used for hyperparameter tuning and early stopping of model training/refinement. For a fair comparison, the data partition on the target dataset used for model training, validation, and testing in the baseline schemes are exactly the same as the data partition used for model refinement, validation, and testing of transfer learning in corresponding cross-validation trials, respectively.

### Ensemble transfer learning scheme

**Figure 2:**
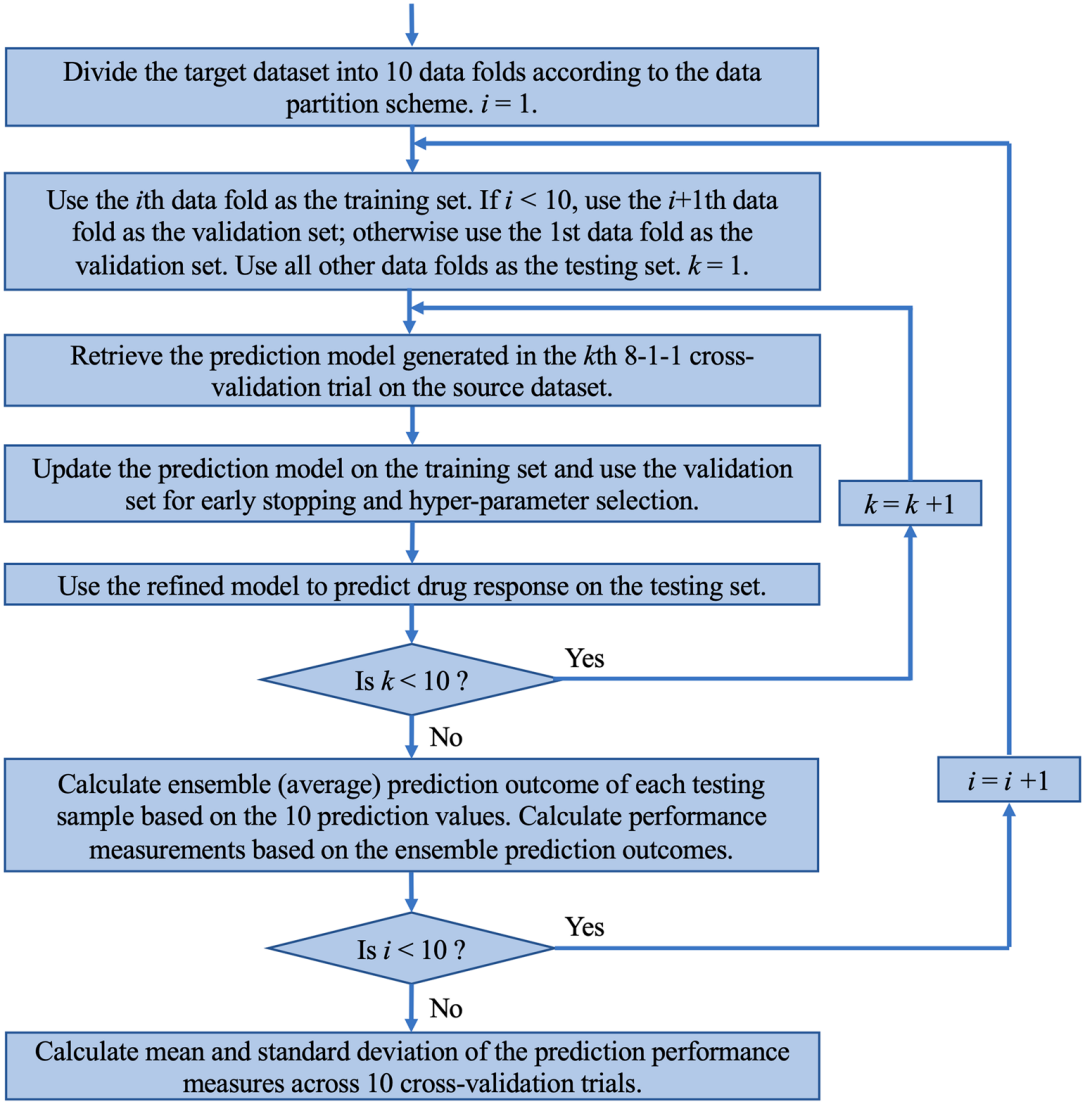
Flowchart of ensemble transfer learning (ETL).

Figure 2 shows the flowchart of ensemble transfer learning (ETL), which retrieves the 10 models trained on the source dataset and refines these models on the training set of the target data. The refined models are then used to predict the testing samples of the target data, where their prediction outcomes are averaged to generate ensemble predictions. We apply the ETL analysis for each of the four transfer learning tasks.

### Three data partition and selection schemes representing different drug response prediction applications

We investigate the power of transfer learning for three different drug response prediction applications including drug repurposing, precision oncology, and new drug development. We design three data partition and selection schemes to simulate the three different applications for transfer learning tasks. For the purpose of evaluating generalization prediction performance, there should be no treatment (combination of CCL and drug) shared by the source and target datasets in analysis. Thus, for each transfer learning task, we removed the overlapping treatments from the source dataset, so that they are included only in the target dataset. For drug repurposing, no additional data removal or selection was performed. For the application of precision oncology, we further removed from the source dataset treatments of CCLs that are also included in the target dataset, because the general goal of precision oncology is to select a drug for treating a tumor that has not been seen before. Also, when performing cross-validations on both the target and source datasets, the data folds were generated to have random but different CCLs, which guaranteed that different CCLs were used for model training/refinement, validation, and testing, strictly simulating the precision oncology setup. For the application of new drug development, we removed from the source dataset treatments of drugs that are also included in the target dataset, because the goal is to discover new drugs that can treat existing cancer cases. When performing cross-validations on both the target and source datasets, the data folds were randomly generated to have different drugs, which guaranteed different drugs were used for model training/refinement, validation, and testing. See Supplementary Table S2 for the numbers of CCLs, drugs, and treatments in the source datasets after data selection for different drug response prediction applications in each transfer learning task.

### DNN and LightGBM prediction models

We take drug response prediction as a regression problem to predict the AUC value and use the mean squared error (MSE) as the loss function to train prediction models. Two different kinds of prediction models, DNN and LightGBM, are used to implement the ETL, SCV, and ECV analyses. LightGBM is an efficient implementation of the Gradient Boosting Decision Tree (GBDT) that has been successfully used in many applications^43–45^. In each boosting step, LightGBM generates a decision tree to fit the negative gradient of loss function with respect to the current prediction, which is a weighted summation of predictions from all previous decision trees. In the case of MSE loss function, the negative gradient is proportional to the prediction residual. After the decision tree is fitted, its prediction outcome is weighted and added to the current prediction to generate a new prediction in the boosting procedure. The learning step size is controlled by a learning rate that can be dynamically changed during the learning process. To prevent overfitting, early stopping of the learning process and regularization on parameters can be applied. Compared to other GBDT algorithms, LightGBM has the advantage of being computationally light for fast model training thanks to the techniques of gradient-based one-side sampling and exclusive feature bundling to speed up model training^43^. To train the LightGBM model, gene expressions and drug descriptors are concatenated to form the input vectors. In transfer learning, the refinement of a LightGBM model was realized by adding additional boosting steps (decision trees) to fit the training set of the target data. See Section 4 of the Supplementary Information for more details of training LightGBM prediction models.

**Figure 3:**
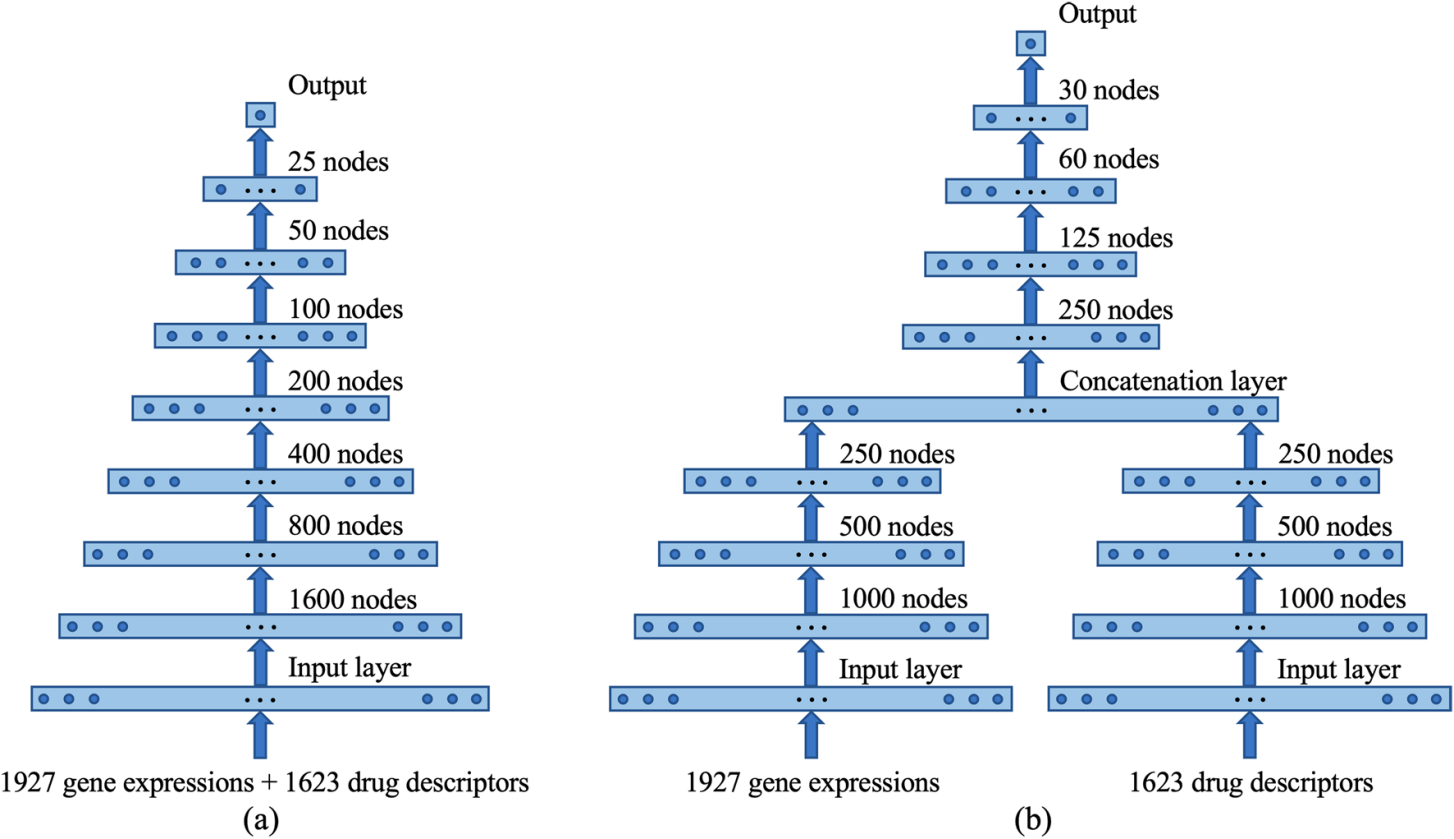
Architectures of two DNN models used in the analysis. (**a**) Single-network DNN (sDNN) model. Gene expressions and drug descriptors are concatenated to form the input. (**b**) Two-subnetwork DNN (tDNN) model. The subnetworks take gene expressions and drug descriptors as separate inputs.

Two DNN models with different architectures were implemented for analysis (see Fig. 3). The first DNN model is composed of seven hidden fully connected (dense) layers with the number of nodes consecutively halved from the first hidden layer to the last hidden layer (Fig. 3a). Gene expressions and drug descriptors are concatenated to form the input. The second DNN model contains two subnetworks of three hidden dense layers, one for the input of gene expressions and the other for the input of drug descriptors (Fig. 3b). The outputs of the two subnetworks are concatenated and then passed to the other four hidden dense layers before output. The number of nodes is also consecutively halved from the first hidden layer to the last hidden layer. For convenience, we use sDNN (single-network DNN) and tDNN (two-subnetwork DNN) to denote the first and second DNN models, respectively. Both sDNN and tDNN have seven hidden layers. Notice that although the total number of nodes in a hidden layer of tDNN is always larger than the number of nodes in the corresponding hidden layer of sDNN, the total number of trainable parameters in tDNN is significantly smaller than that of sDNN due to the subnetwork structure. In both networks, each hidden layer has a dropout layer following it except the last hidden layer. When refining a trained DNN model on the target dataset for transfer learning, we kept the parameters of the bottom two hidden layers unchanged and continued training the parameters associated with the top five hidden layers on the target dataset. See Section 4 of the Supplementary Information for details of training DNN prediction models.

## Results

For each of the three drug response prediction applications, we performed the analyses of ensemble transfer learning (ETL), standard cross-validation (SCV), and ensemble cross-validation (ECV) with three prediction models including LightGBM, sDNN (single-network DNN), and tDNN (two-subnetwork DNN). ETL was conducted for four transfer learning tasks including CTRP → CCLE, CTRP → GCSI, GDSC → CCLE, and GDSC → GCSI. Thus, a total number of 3 $\times$ 3 $\times$ 4 = 36 transfer learning analyses were conducted. SCV and ECV were conducted on the two target datasets, CCLE and GCSI. The total numbers of SCV and ECV analyses are both 3 $\times$ 3 $\times$ 2 = 18. We used two measures to evaluate the testing prediction performance. The first measure is the root of mean squared error (RMSE), which is the square root of the loss function optimized by the prediction models. The second measure is the Pearson correlation coefficient between prediction values and true values. The prediction performance was evaluated 10 times in the 10 cross-validation trials for each of ETL, SCV, and ECV. To rigorously evaluate whether ETL can improve the prediction performance, we always compared the prediction performance of ETL to that of SCV/ECV on the same target dataset and with the same prediction model. The statistical significance of the difference between the prediction performances of ETL and SCV/ECV was evaluated using the pair-wise two-tail t test^46^, based on the 10 performance measurements obtained in cross-validations for each analysis scheme.

### Prediction performance for drug repurposing application

Table 1 shows the obtained prediction performance and comparison for the drug repurposing application. Each row in Table 1 is for the comparison of ETL to SCV and ECV on one target dataset and with one prediction model. Every three adjacent rows are for one transfer learning task with the same pair of source and target datasets, but with different prediction models used for analysis. RMSE related results are in columns 4–8 and results related to Pearson correlation coefficient (denoted by Cor in Table 1) are in columns 9–13. In all of the 12 comparisons (rows in Table 1), ETL always outperforms SCV and ECV, indicated by both smaller average RMSE and larger average correlation coefficients. T-tests also show that the performance improvement of ETL is always statistically significant (p-values ≤ 0.05). This demonstrates the benefit of using ensemble transfer learning for drug response prediction in drug repurposing application. In Table 1, the best prediction performance achieved for each transfer learning task is indicated in bold. Compared across the three different prediction models, ETL with tDNN outperforms ETL with the other two prediction models in all four transfer learning tasks, also indicated by both smaller average RMSE and larger average correlation coefficients. When applied on the same target dataset with the same prediction model, ECV always gives an improved average RMSE and correlation coefficient compared to SCV, which is consistent with the expectation that ensemble learning is often beneficial.

**Table 1:** Comparison on the prediction performance of standard cross-validation (SCV), ensemble cross-validation (ECV), and ensemble transfer learning (ETL) for drug repurposing application.

Target	Source	Model	RMSE (SCV)	RMSE (ECV)	RMSE (ETL)	P-value (RMSE, SCV vs. ETL)	P-value (RMSE, ECV vs. ETL)	Cor (SCV)	Cor (ECV)	Cor (ETL)	P-value (Cor, SCV vs. ETL)	P-value (Cor, ECV vs. ETL)
CCLE	CTRP	LightGBM	0.0895 (0.0007)	0.0872 (0.0009)	0.0827 (0.0007)	2.30E−11	1.36E−08	0.8313 (0.0029)	0.8403 (0.0037)	0.8581 (0.0023)	4.30E−11	1.82E−08
		sDNN	0.0895 (0.0013)	0.0863 (0.0010)	0.0812 (0.0007)	3.13E−08	4.17E−07	0.8341 (0.0050)	0.8466 (0.0045)	0.8672 (0.0030)	1.97E−08	4.72E−07
		tDNN	0.0918 (0.0009)	0.0867 (0.0009)	**0.0756** (0.0005)	4.96E−12	7.66E−11	0.8236 (0.0033)	0.8435 (0.0030)	**0.8841** (0.0025)	2.85E−12	3.81E−11
CCLE	GDSC	LightGBM	0.0895 (0.0007)	0.0872 (0.0009)	0.0839 (0.0009)	5.06E−09	2.52E−07	0.8313 (0.0029)	0.8403 (0.0037)	0.8535 (0.0035)	8.89E−10	3.13E−08
		sDNN	0.0895 (0.0013)	0.0863 (0.0010)	0.0838 (0.0008)	3.71E−07	2.43E−06	0.8341 (0.0050)	0.8466 (0.0045)	0.8562 (0.0037)	6.10E−07	1.42E−05
		tDNN	0.0918 (0.0009)	0.0867 (0.0009)	**0.0811** (0.0007)	2.85E−10	9.30E−08	0.8236 (0.0033)	0.8435 (0.0030)	**0.8654** (0.0022)	1.25E−10	1.55E−08
GCSI	CTRP	LightGBM	0.1168 (0.0005)	0.1142 (0.0007)	0.1063 (0.0015)	2.08E−09	3.89E−08	0.7889 (0.0018)	0.7992 (0.0017)	0.8293 (0.0048)	2.11E−10	3.85E−09
		sDNN	0.1167 (0.0025)	0.1119 (0.0017)	0.1051 (0.0014)	7.05E−07	1.57E−06	0.7956 (0.0111)	0.8118 (0.0057)	0.8384 (0.0047)	2.13E−06	1.26E−06
		tDNN	0.1177 (0.0032)	0.1109 (0.0014)	**0.0962** (0.0018)	7.93E−09	4.92E−09	0.7923 (0.0105)	0.8133 (0.0050)	**0.8633** (0.0055)	1.72E−08	4.97E−09
GCSI	GDSC	LightGBM	0.1168 (0.0005)	0.1142 (0.0007)	0.1059 (0.0015)	1.99E−09	4.93E−08	0.7889 (0.0018)	0.7992 (0.0017)	0.8321 (0.0056)	7.83E−10	7.97E−09
		sDNN	0.1167 (0.0025)	0.1119 (0.0017)	0.1047 (0.0021)	4.92E−06	1.57E−05	0.7956 (0.0111)	0.8118 (0.0057)	0.8419 (0.0048)	2.43E−06	9.13E−07
		tDNN	0.1177 (0.0032)	0.1109 (0.0014)	**0.0995** (0.0017)	1.76E−09	1.12E−09	0.7923 (0.0105)	0.8133 (0.0050)	**0.854** (0.0052)	1.54E−09	9.77E−11

RMSE indicates the square root of mean square error. Cor indicates the Pearson correlation coefficient. In the RMSE and Cor columns, the number before a parenthesis is the average prediction performance and the number in a parenthesis is the standard deviation, calculated across 10 cross-validation trials. The p-values are generated by t-tests and indicate how significantly the prediction performance of ETL differs from those of SCV and ECV. SCV vs. ETL indicates comparison of SCV and ETL. ECV vs. ETL indicates comparison of ECV and ETL. The best average prediction performance for each transfer learning task is indicated with bold.

### Prediction performance for precision oncology application

Table 2 shows the prediction performance and comparison for the precision oncology application, with cross-validations based on hard partitioning of CCLs. The arrangement of results and comparisons in Table 2 follows the style of Table 1. Each row in Table 2 is for the comparison of ETL to SCV and ECV on one target dataset and with one prediction model, and every three adjacent rows are for one transfer learning task with different prediction models. In all four transfer learning tasks and with all three prediction models, ETL almost always statistically significantly (p-values ≤ 0.05) outperforms SCV and ECV with improved average RMSE and correlation coefficients, which indicates the benefit of using ensemble transfer learning for drug response prediction in precision oncology. The only exception occurs when sDNN model is used for the GDSC → CCLE transfer learning task. Compared between different prediction models, ETL with tDNN always outperforms ETL with the other two prediction models, LightGBM and sDNN, except only in the CTRP → CCLE transfer learning task when the prediction performance is evaluated by the correlation coefficient. Again, when applied on the same target dataset with the same prediction model, ECV always gives a better prediction performance than SCV does, demonstrating the benefit of ensemble learning.

**Table 2:** Comparison on the prediction performance of standard cross-validation (SCV), ensemble cross-validation (ECV), and ensemble transfer learning (ETL) for precision oncology application.

Target	Source	Model	RMSE (SCV)	RMSE (ECV)	RMSE (ETL)	P-value (RMSE, SCV vs. ETL)	P-value (RMSE, ECV vs. ETL)	Cor (SCV)	Cor (ECV)	Cor (ETL)	P-value (Cor, SCV vs. ETL)	P-value (Cor, ECV vs. ETL)
CCLE	CTRP	LightGBM	0.0913 (0.0015)	0.0894 (0.0015)	0.087 (0.0016)	6.43E−06	1.08E−04	0.8245 (0.0045)	0.8325 (0.0047)	0.8419 (0.0056)	3.75E−06	8.75E−05
		sDNN	0.0915 (0.0014)	0.0886 (0.0009)	0.0858 (0.0014)	5.99E−06	5.27E−07	0.8275 (0.0052)	0.8385 (0.0041)	**0.8479** (0.0049)	3.67E−06	2.90E−05
		tDNN	0.0909 (0.0013)	0.0882 (0.0009)	**0.0856** (0.0014)	2.39E−06	3.89E−05	0.8293 (0.0040)	0.8386 (0.0038)	0.8476 (0.0037)	2.69E−06	1.46E−04
CCLE	GDSC	LightGBM	0.0913 (0.0015)	0.0894 (0.0015)	0.0877 (0.0014)	5.37E−07	1.58E−04	0.8245 (0.0045)	0.8325 (0.0047)	0.8389 (0.0045)	6.01E−07	1.80E−04
		sDNN	0.0915 (0.0014)	0.0886 (0.0009)	0.0888 (0.0013)	3.27E−04	3.28E−01	0.8275 (0.0052)	0.8385 (0.0041)	0.8366 (0.0038)	1.26E−05	4.86E−03
		tDNN	0.0909 (0.0013)	0.0882 (0.0009)	**0.0869** (0.0012)	2.55E−05	3.87E−03	0.8293 (0.0040)	0.8386 (0.0038)	**0.8428** (0.0040)	1.22E−04	1.07E−02
GCSI	CTRP	LightGBM	0.1186 (0.0023)	0.116 (0.0026)	0.1118 (0.0029)	7.89E−05	1.75E−03	0.783 (0.0090)	0.7929 (0.0094)	0.8087 (0.0109)	9.27E−05	1.69E−03
		sDNN	0.123 (0.0043)	0.1218 (0.0033)	0.1118 (0.0016)	1.25E−05	9.55E−06	0.7798 (0.0160)	0.7938 (0.0082)	0.8119 (0.0049)	1.14E−04	5.43E−05
		tDNN	0.1237 (0.0043)	0.118 (0.0029)	**0.1085** (0.0012)	3.36E−06	5.19E−06	0.7804 (0.0084)	0.7989 (0.0083)	**0.8228** (0.0042)	2.83E−07	2.44E−05
GCSI	GDSC	LightGBM	0.1186 (0.0023)	0.116 (0.0026)	0.1099 (0.0020)	8.89E−08	6.55E−06	0.783 (0.0090)	0.7929 (0.0094)	0.8162 (0.0072)	1.59E−07	7.10E−06
		sDNN	0.123 (0.0043)	0.1218 (0.0033)	0.1106 (0.0016)	6.42E−06	3.48E−06	0.7798 (0.0160)	0.7938 (0.0082)	0.8156 (0.0063)	3.76E−05	6.37E−07
		tDNN	0.1237 (0.0043)	0.118 (0.0029)	**0.1076** (0.0015)	2.34E−06	6.80E−07	0.7804 (0.0084)	0.7989 (0.0083)	**0.8258** (0.0046)	4.06E−08	2.86E−07

RMSE indicates the square root of mean square error. Cor indicates the Pearson correlation coefficient. In the RMSE and Cor columns, the number before a parenthesis is the average prediction performance and the number in a parenthesis is the standard deviation, calculated across 10 cross-validation trials. The p-values are generated by t-tests and indicate how significantly the prediction performance of ETL differs from those of SCV and ECV. SCV vs. ETL indicates comparison of SCV and ETL. ECV vs. ETL indicates comparison of ECV and ETL. The best average prediction performance for each transfer learning task is indicated with bold.

### Prediction performance for new drug development application

Table 3 shows the prediction performance and comparison for the new drug development application with cross-validations based on hard partitioning of drugs. The arrangement of results and comparisons in Table 3 follows the style of Tables 1 and 2. Predicting the efficacy of new drugs not included in the training set is generally a more challenging task than predicting the response of new CCLs. Also, because there are not many drugs tested in the CCLE and GCSI studies (see Supplementary Table S1), the number of drugs used for training or refining a prediction model on these two target datasets is no larger than three, which forms a very difficult prediction problem. It is not surprising to see that the prediction performance of ETL is worse for new drug development than for precision oncology and drug repurposing. But ETL’s improvement on the prediction performance over ECV/SCV, which is evaluated by the difference between the prediction performances of ETL and ECV/SCV, is also higher for new drug development than for the other two applications.

**Table 3:** Comparison on the prediction performance of standard cross-validation (SCV), ensemble cross-validation (ECV), and ensemble transfer learning (ETL) for the application of new drug development.

Target	Source	Model	RMSE (SCV)	RMSE (ECV)	RMSE (ETL)	P-value (RMSE, SCV vs. ETL)	P-value (RMSE, ECV vs. ETL)	Cor (SCV)	Cor (ECV)	Cor (ETL)	P-value (Cor, SCV vs. ETL)	P-value (Cor, ECV vs. ETL)
CCLE	CTRP	LightGBM	0.1828 (0.0249)	0.1826 (0.0249)	0.1589 (0.0125)	2.32E−02	2.42E−02	0.0739 (0.0781)	0.0778 (0.0815)	0.3742 (0.1490)	1.52E−04	1.45E−04
		sDNN	0.2132 (0.0608)	0.1964 (0.0460)	0.158 (0.0152)	3.20E−02	4.52E−02	0.0762 (0.0803)	0.0685 (0.1638)	0.4455 (0.0965)	3.77E−05	8.99E−04
		tDNN	0.206 (0.0637)	0.205 (0.0602)	**0.1553** (0.0176)	4.98E−02	4.46E−02	0.0917 (0.1589)	0.0937 (0.1446)	**0.4667** (0.1172)	1.20E−04	6.99E−05
CCLE	GDSC	LightGBM	0.1828 (0.0249)	0.1826 (0.0249)	**0.1283** (0.0053)	9.11E−05	9.57E−05	0.0739 (0.0781)	0.0778 (0.0815)	**0.6301** (0.0525)	2.52E−09	2.80E−09
		sDNN	0.2132 (0.0608)	0.1964 (0.0460)	0.146 (0.0201)	1.04E−02	8.23E−03	0.0762 (0.0803)	0.0685 (0.1638)	0.5717 (0.0539)	5.76E−08	8.65E−06
		tDNN	0.206 (0.0637)	0.205 (0.0602)	0.1412 (0.0214)	2.28E−02	1.92E−02	0.0917 (0.1589)	0.0937 (0.1446)	0.6124 (0.0638)	1.09E−05	6.65E−06
GCSI	CTRP	LightGBM	0.2491 (0.0402)	0.249 (0.0401)	**0.1975** (0.0197)	3.03E−03	3.02E−03	0.163 (0.0643)	0.1707 (0.0599)	**0.396** (0.0366)	1.41E−05	1.54E−05
		sDNN	0.2804 (0.0584)	0.3042 (0.0693)	0.2243 (0.0346)	1.54E−02	1.57E−02	0.0172 (0.2006)	− 0.2031 (0.1689)	0.2231 (0.1606)	8.78E−02	1.27E−03
		tDNN	0.3043 (0.0931)	0.2988 (0.0670)	0.215 (0.0348)	1.97E−02	6.88E−03	− 0.1835 (0.1726)	− 0.1463 (0.2150)	0.3707 (0.0751)	8.90E−06	1.17E−04
GCSI	GDSC	LightGBM	0.2491 (0.0402)	0.249 (0.0401)	**0.2075** (0.0268)	8.21E−03	8.23E−03	0.163 (0.0643)	0.1707 (0.0599)	**0.3878** (0.0513)	3.11E−05	3.63E−05
		sDNN	0.2804 (0.0584)	0.3042 (0.0693)	0.2255 (0.0239)	2.42E−02	8.93E−03	0.0172 (0.2006)	− 0.2031 (0.1689)	0.1092 (0.2477)	4.89E−01	2.36E−03
		tDNN	0.3043 (0.0931)	0.2988 (0.0670)	0.2148 (0.0295)	2.13E−02	7.11E−03	− 0.1835 (0.1726)	− 0.1463 (0.2150)	0.3147 (0.0983)	5.37E−06	2.87E−04

RMSE indicates the square root of mean square error. Cor indicates the Pearson correlation coefficient. In the RMSE and Cor columns, the number before a parenthesis is the average prediction performance and the number in a parenthesis is the standard deviation, calculated across 10 cross-validation trials. The p-values are generated by t-tests and indicate how significantly the prediction performance of ETL differs from those of SCV and ECV. SCV vs. ETL indicates comparison of SCV and ETL. ECV vs. ETL indicates comparison of ECV and ETL. The best average prediction performance for each transfer learning task is indicated with bold.

In all four transfer learning tasks and with all three prediction models, ETL always outperforms SCV and ECV, demonstrated by smaller average RMSE and higher average correlation coefficients. ETL’s improvement on prediction performance is always statistically significant (p-values ≤ 0.05), except only in the comparison of ETL and SCV on the GCSI dataset when sDNN is the prediction model used for analysis and the prediction performance is evaluated by the correlation coefficient. This result indicates the benefit of using ensemble transfer learning for new drug development. Compared among the three prediction models, ETL with tDNN performs best in the transfer learning task of CTRP → CCLE, while ETL with LightGBM performs best in the other three transfer learning tasks. This is different from the cases of drug repurposing and precision oncology, where ETL with tDNN almost always outperforms ETL with LightGBM or sDNN. A possible reason is that LightGBM has a model complexity lower than those of DNN models, measured by the number of trainable parameters. Thus, it is more generalizable for predicting the efficacy of new drugs, especially when the training data include very few drugs.

### Prediction performance of transfer learning using individual model without ensemble

Since we have performed ensemble transfer learning, it is straightforward to calculate the prediction performance of transfer learning using an individual model without ensemble prediction, which is called standard transfer learning (STL). Detail results of STL cannot be presented due to the large number of models trained in the analysis, but we can summarize here the major observation based on the results. In the drug repurposing and precision oncology applications, STL sometimes does not produce a prediction performance better than those of SCV and ECV. On the contrary, as we have presented in the previous subsections, ETL dominantly outperforms SCV and ECV for these two applications, which indicates the importance of using transfer learning and ensemble prediction simultaneously for drug response prediction. For the more challenging application of new drug development, we find STL almost always outperforms SCV and ECV, while ETL further improves the prediction performance compared to STL. ETL, STL, SCV, and ECV are always compared based on the same target dataset and the same prediction model for fairness.

## Discussion

We developed the first ensemble transfer learning framework for building general prediction models of anti-cancer drug response. The transfer learning pipeline was implemented with three different prediction models including LightGBM, sDNN (single-network DNN), and tDNN (two-subnetwork DNN). We designed a comprehensive evaluation scenario to investigate the performance of the transfer learning pipeline for three different drug response prediction applications, including drug repurposing, precision oncology, and new drug development, based on in vitro drug screening datasets. Our analysis results demonstrate the benefit of applying ensemble transfer learning in all of the three applications. For the comparison between transfer learning implemented with different prediction models, ETL with tDNN performs best in the drug repurposing and precision oncology applications, while ETL with LightGBM outperforms the other two models in three out of the four transfer learning tasks for new drug development.

Compared with existing works, our study is the first research attempt of its kind with unique contributions, which can be summarized from three aspects. First, while existing transfer learning studies for drug response prediction all focus on building drug-specific prediction models, we target the more challenging task of building general drug response prediction models that are not specific to a drug. Our study is the first one to show transfer learning can improve the performance of general drug response prediction models. This result indicates the potential of improving existing drug response prediction methods by designing and applying appropriate transfer learning procedures. Second, we study the power of transfer learning and show its advantage in three different drug response prediction applications including drug repurposing, precision oncology, and new drug development, which to our knowledge has not been investigated before. Our analysis design gives an example for future studies that need to evaluate the performance of drug response prediction in different application setups. Third, unlike previous transfer learning studies that emphasize building transformations of features and drug response values between datasets^39^, our proposed ETL framework applies the classic transfer learning scheme and extends it through ensemble, which trains multiple prediction models on the source data and then refine them on the target data for ensemble prediction. Although there usually exist considerable variations between different drug screening studies/datasets^39^, ETL with model refinement and ensemble prediction on the target dataset seems to overcome this gap and extract useful information from the source dataset to construct prediction models on the target dataset.

Our main goal is to develop a general transfer learning framework that is insensitive to the underlying machine learning methods for building general drug response prediction models. For this reason, we pick three representative prediction models to implement the proposed ETL framework and demonstrate its ability of improving the performance of all three models. We choose LightGBM, an efficient GBDT method, to represent the conventional machine learning algorithms, as GBDT models have been successfully used in many applications^43–45^. Compared to other GBDT algorithms, LightGBM also has the advantage of being computationally light for fast model training^43^. For deep learning models, because whether the two input data modalities (gene expressions and drug descriptors) are concatenated to form the input vector or separately input into subnetworks makes a significant difference on the number of trainable parameters (i.e. model complexity), we choose to test both sDNN and tDNN. To keep the hidden layers in the network models representative and generic, we use the fully connected dense layers. In transfer learning with the DNN models, we also tried freezing the parameters of the bottom four hidden layers and adjusting the parameters associated with the top three hidden layers and the dropout rate in the model refinement stage. The obtained prediction performance was worse than what we got when freezing only the bottom two hidden layers, indicating the importance of having sufficient layers trainable for model refinement in transfer learning.

For the transfer learning tasks, we use the CTRP and GDSC datasets separately as two source datasets rather than combine them to form one source dataset. The reason is two-fold. First, datasets generated in different drug screening studies are usually heterogenous^39^, which makes it challenging to combine them without introducing additional bias. Differences in experimental protocols, assays, or biological models and drugs used in the studies generate variations between these datasets. Specifically, CTRP used the CellTiterGlo assay to measure cell viability, while GDSC used the Resazurin and Syto60 assays. Second, using CTRP and GDSC datasets separately gives us four transfer learning tasks rather than two, providing us more opportunities to test and evaluate the proposed ETL framework.

Although our current work successfully demonstrates the benefit of applying ETL for building general drug response prediction models, there are three potential limitations indicating important research directions in future work. First, our study focuses on predicting the efficacy of single-drug treatments, while it is also an important task to predict the efficacy of drug combinations^11,21–23^. Although methods have been proposed for predicting the efficacy of drug combinations^11, 21–23^, transfer learning has not been explored for improving the prediction performance in this task. We plan to investigate transfer learning for building prediction models of drug combinations. Prediction patterns learned on a single-drug screening dataset or a drug combination screening dataset can be transferred to another drug combination screening study for building prediction models. Second, while our current study implements the proposed ETL framework with three prediction models/algorithms, it has the potential to be implemented with many other prediction algorithms. Successful applications of ETL require updating the prediction models based on the target domain data, which adapts the models to the target prediction tasks. In the future, proper model refinement procedures need to be researched for various kinds of prediction algorithms to apply transfer learning. Third, our current transfer learning study between in vitro drug screening datasets is only a pilot effort to guide future application of transfer learning to improve drug response prediction performance on patients or patient derived models, such as xenografts (PDXs)^47^ and organoids (PDOs)^48^. The ultimate goal of predicting drug response is to either recommend an existing drug or design a new drug for treating a cancer patient. Biological models, such as CCLs, PDXs, and PDOs, are different from each other and also different from the real patient tumors, leading to the variations of their drug responses. Transfer learning provides a promising way to utilize drug response information of one biological model to help predict the drug response of another biological model. For example, transfer learning utilizing the relatively abundant in vitro drug screening data to help predict drug response in PDXs, PDOs, and eventually in patients with limited data will be important in future research.

## Supplementary information

Supplementary Information
