# Supplementary material for: Ensemble transfer learning for the prediction of anti-cancer drug response
Source: Sci Rep. 2020 Oct 22;10:18040. doi: 10.1038/s41598-020-74921-0 (PMC7581765; doi:10.1038/s41598-020-74921-0)
Supplement: Supplementary file 1 — Supplementary Information [file 41598_2020_74921_MOESM1_ESM.pdf]

Ensemble Transfer Learning for the Prediction of Anti-Cancer Drug Response

(Supplementary Information)

Yitan Zhu[1*], Thomas Brettin[1], Yvonne A. Evrard[2], Alexander Partin[1], Fangfang Xia[1], Maulik Shukla[1], Hyunseung Yoo[1], James H. Doroshow[3], Rick L. Stevens[1,4]

1. Computing, Environment and Life Sciences, Argonne National Laboratory, Lemont, IL 60439, USA

2. Frederick National Laboratory for Cancer Research, Leidos Biomedical Research, Inc. Frederick, MD 21702, USA

3. Developmental Therapeutics Branch, National Cancer Institute, Bethesda, MD 20892, USA

4. Department of Computer Science, The University of Chicago, Chicago, IL 60637, USA

[*]Correspondence: yitan.zhu@anl.gov

Section 1: Drug Response Data, Gene Expressions, and Drug Descriptors

Our study involves four public in vitro drug screening datasets, including the Cancer Therapeutics Response Portal v2 (CTRP)[1], the Genomics of Drug Sensitivity in Cancer (GDSC)[2], the Cancer Cell Line Encyclopedia (CCLE)[3], and the Genentech Cell Line Screening Initiative (GCSI)[4]. The drug response values of these datasets are the percentages of tumor cell growth under a drug treatment at multiple doses. We used the three-parameter logistic function (hill slope model) to fit the tumor cell growth values and generate dose response curves. Based on the dose response curve, we calculated the area under the dose response curve (AUC) for the dose range of [10^{-10} M, 10^{-4} M]. The AUC value was then normalized by the dose range, so that after normalization, the AUC value is between 0 and 1, representing the treatment effect. 0 indicates complete response and 1 indicates no response. Supplementary Table 1 shows the number of treatments (pairs of drugs and cancer cell lines) in each dataset. In a study, a drug and cancer cell line (CCL) pair may have been tested multiple times. In these cases, we averaged the AUC values across the experiments so that the number of treatments in Supplementary Table 1 reflects the number of unique drug and CCL pairs in a dataset. Supplementary Fig. 1 shows the histogram of AUC values in each dataset with the mean and standard deviation calculated. Clearly, the distribution of AUC values varies between datasets.

Supplementary Table 1 Numbers of CCLs, drugs, and treatments (pairs of drugs and CCLs) in each dataset.

Dataset	# CCLs	# Drugs	# Treatments
GCSI	357	16	5,647
CCLE	474	24	10,971
GDSC	659	238	125,712
CTRP	812	494	318,040

CCLs are represented by their gene expressions in prediction modeling. The gene expression data were collected from the CCLE online resource. All CCLs in the other three studies, i.e., GCSI, CTRP, and GDSC, were also used in the CCLE study, except 11 GDSC CCLs that were thus excluded from the analysis. The gene expression data were generated using RNA sequencing, and TPMs (transcripts per kilobase million) were calculated as expression values, which were log2 transformed and then standardized so that each gene has a 0 mean and a unit standard deviation. Instead of using all transcripts for analysis, we focused the analysis on genes potentially related to cancer genetic mechanism, genomic regulation, and drug response. We selected 1,927 genes, including "landmark" genes well-representing cellular transcriptomic changes identified in the Library of Integrated Network-Based Cellular Signatures (LINCS) project[5] and cancer-related genes collected from OncoKB[6] and GDSC[7].

Drugs are represented by molecular descriptors in prediction modeling. The Dragon (version 7.0) software package (https://chm.kode-solutions.net/products_dragon.php) was used to

compute numeric descriptors of the drugs based on their molecular structure. The package calculated various types of descriptors, such as the simplest atom types, functional groups and fragment counts, topological and geometrical descriptors, estimations of molecular properties, and drug-like and lead-like indices. We removed the descriptors with missing values and kept 1,623 molecular descriptors for the analysis.

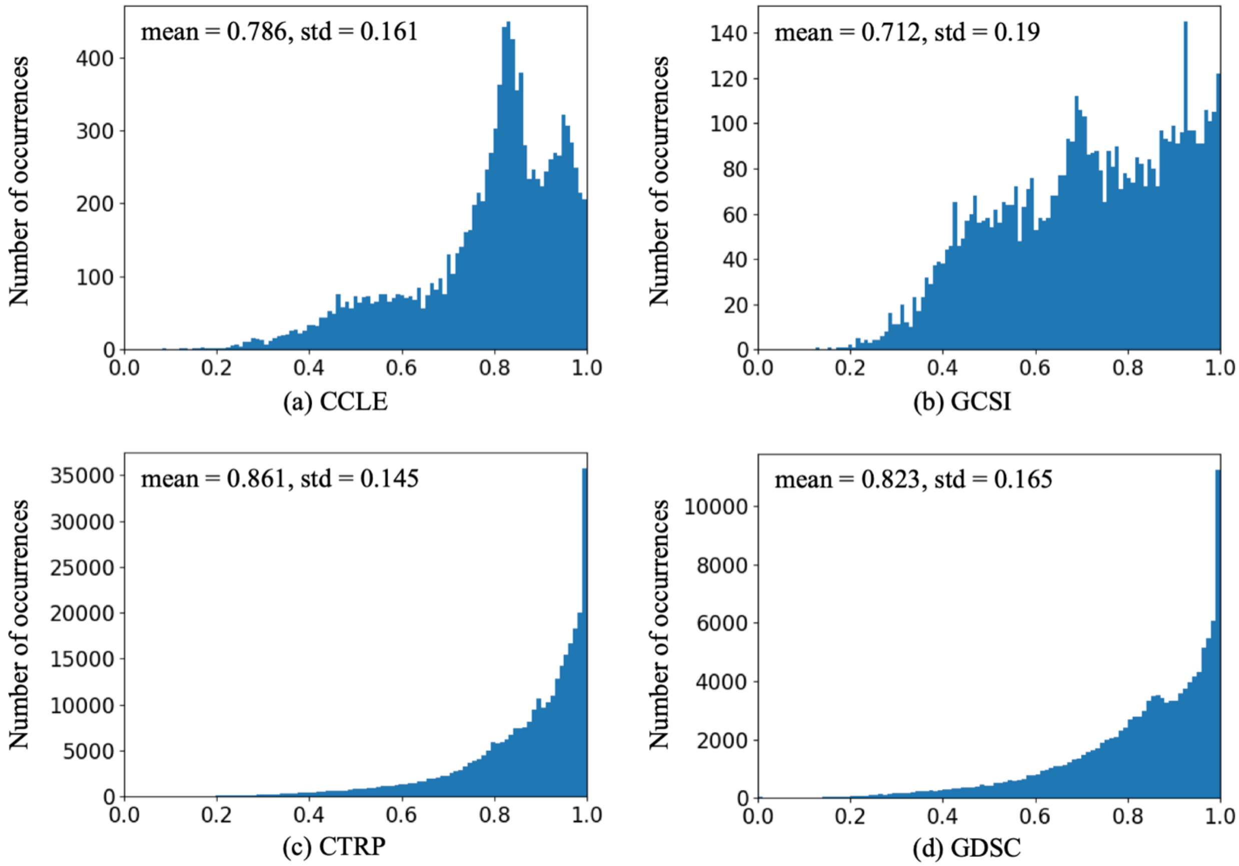

Supplementary Figure 1 Histograms of drug response AUC values in datasets. Mean and standard deviation (std) of AUC values are shown on the top left of each histogram.

Section 2: Schemes of Standard and Ensemble Cross-Validations

Supplementary Fig. 2a shows the flowchart of standard cross-validation (SCV), in which data are divided into three parts for model training, validation, and testing. 8-1-1 cross-validation means dividing the data into 10 data folds and using 8, 1, and 1 data fold for model training, validation, and testing, respectively. 8-1-1 cross-validation is used at the first step of transfer learning to train models on the source dataset. 1-1-8 cross-validation means dividing the data into 10 data folds and using 1, 1, and 8 data folds for model training/refinement, validation, and testing,

respectively. 1-1-8 cross-validation is used for all analyses on the target data, to simulate a situation where the training data at the target domain are quite limited. The 1-1-8 SCV is applied on the target data as a baseline to be compared with ensemble transfer learning (ETL). The ensemble cross-validation (ECV) also follows the flowchart in Supplementary Fig. 2a but with the part indicated by the dashed-line box replaced by the flowchart shown in Supplementary Fig. 2b, which performs ensemble learning by resampling the training set. We apply the 1-1-8 ECV on the target data as a second baseline to be compared with ETL. Notice that SCV, ECV, and ETL on the same target dataset always use the same data partition (i.e., training/refinement, validation, and testing sets) in corresponding cross-validation trials, so that the prediction performances obtained by the analyses can be compared.

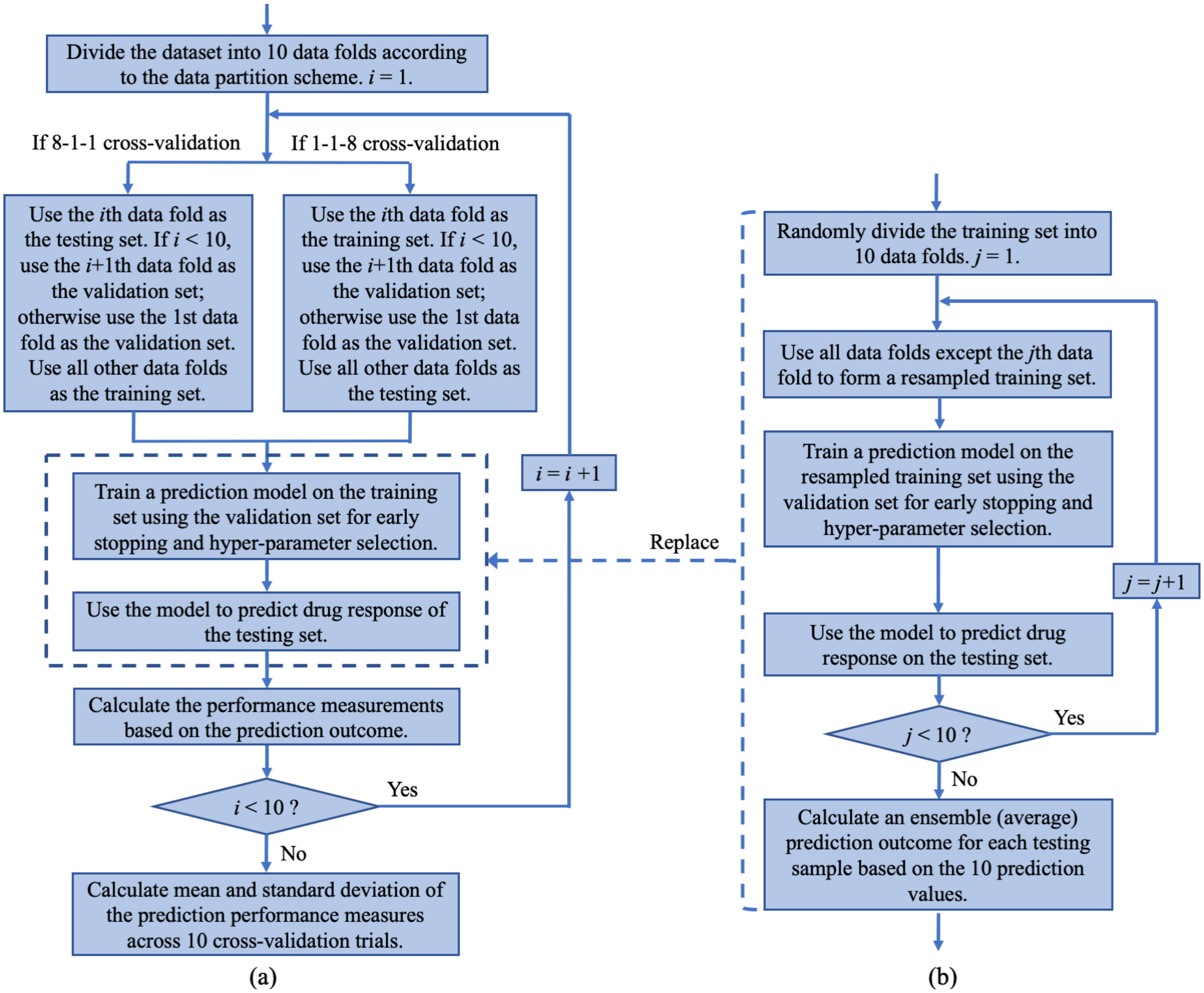

Supplementary Figure 2 (a) Flowchart of standard cross-validation (SCV). (b) The ensemble cross-validation (ECV) also follows the flowchart in (a), but with the part indicated by the dashed-line box replaced by the flowchart in (b).

Section 3: Information About Source Datasets After Data Selection

We investigate four transfer learning tasks for three different drug response prediction applications including drug repurposing, precision oncology, and new drug development. We design and apply three data partition and selection schemes to simulate the three applications as described in the main text. Supplementary Table 2 shows the numbers of CCLs, drugs, and treatments in the source datasets after applying the data selection schemes. Section B of Supplementary Table 2 shows that the numbers of CCLs and treatments are significantly reduced after the removal of overlap CCLs for the precision oncology application compared to the numbers in Sections A and B for drug repurposing and new drug development.

Supplementary Table 2. Numbers of CCLs, drugs, and treatments in source datasets after removing overlap between source and target datasets with different data partition and selection schemes.

Transfer learning task		Section A: removal of overlap treatments for drug repurposing			Section B: removal of overlap CCLs for precision oncology			Section C: removal of overlap drugs for new drug development		
Target	Source	# CCLs	# Drugs	# Treatments	# CCLs	# Drugs	# Treatments	# CCLs	# Drugs	# Treatments
CCLE	CTRP	812	494	311,194	376	494	143,634	812	477	305,278
CCLE	GDSC	659	238	123,447	282	238	53,743	659	225	121,174
GCSI	CTRP	812	494	314,469	479	494	185,138	812	482	309,363
GCSI	GDSC	659	238	123,141	343	238	64,664	659	224	120,045

Section 4: Details of Training DNN and LightGBM Prediction Models

To implement the ensemble transfer learning framework with LightGBM models, we used the LightGBM Python package (https://LightGBM.readthedocs.io/en/latest/index.html). In transfer learning, the refinement of a LightGBM model was realized by adding additional boosting steps (decision trees) to fit the training set of the target data. The model training/refinement process would be stopped early if the loss on the validation set did not reduce in 150 boosting steps; otherwise the whole process took 1,500 boosting steps. For the other parameters of the LightGBM model, we used the default values.

We used the Keras package (https://keras.io/) with Tensorflow (https://www.tensorflow.org/) backend for implementing deep neural network (DNN) models. All dropout layers in a DNN model use the same dropout rate. In the analysis, the dropout rate was selected among 0, 0.1, 0.25, 0.45, and 0.7 by minimizing the validation loss. It was the only hyperparameter optimized in the model learning process. The Adam optimizer was used with default setting for model learning[8]. The learning rate was initialized at 0.001 and was reduced by a factor of 10 if the reduction of validation loss was smaller than 0.00001 in 10 epochs. The

learning process would be early stopped if the reduction of validation loss was smaller than 0.00001 in 20 epochs; otherwise the full learning process would take 100 epochs.

When refining a trained DNN model for transfer learning, we kept the parameters of the bottom 2 hidden layers unchanged and continued training the parameters associated with the top 5 hidden layers on the target dataset. The dropout rate was also re-selected among the five candidate values based on the validation loss. The idea behind this DNN transfer learning approach is that the DNN model trained on the source data forms an iterative and continuous feature abstraction process, in which the bottom layers may generate features that are more generic and informative in both the source and target domains. In the model refining stage on the target data, the parameters of the bottom hidden layers are kept unchanged, so that they serve as a feature extractor to generate the same kind of features as they have done when the model is trained on the source data. The model refinement updates the parameters of the top layers of the DNN models, so that the more abstracted, up-level features can be adapted to the target data.

References

1 Basu, A. *et al.* An interactive resource to identify cancer genetic and lineage dependencies targeted by small molecules. *Cell* **154**, 1151-1161. https://doi.org/10.1016/j.cell.2013.08.003 (2013).

2 Yang, W. *et al.* Genomics of Drug Sensitivity in Cancer (GDSC): a resource for therapeutic biomarker discovery in cancer cells. *Nucleic Acids Res.* **41**, D955-961. https://doi.org/10.1093/nar/gks1111 (2013).

3 Barretina, J. *et al.* The Cancer Cell Line Encyclopedia enables predictive modelling of anticancer drug sensitivity. *Nature* **483**, 603-607. https://doi.org/10.1038/nature11003 (2012).

4 Haverty, P. *et al.* Reproducible pharmacogenomic profiling of cancer cell line panels. *Nature* **533**, 333-337. https://doi.org/10.1038/nature17987 (2016).

5 Subramanian, A. *et al.* A next generation connectivity map: L1000 platform and the first 1,000,000 profiles. *Cell* **171**, 1437-1452.e1417. https://doi.org/10.1016/j.cell.2017.10.049 (2017).

6 Chakravarty, D. *et al.* OncoKB: a precision oncology knowledge base. *JCO Precis. Oncol.* **2017**. https://doi.org/10.1200/PO.17.00011 (2017).

7 Iorio, F. *et al.* A landscape of pharmacogenomic interactions in cancer. *Cell* **166**, 740-754. https://doi.org/10.1016/j.cell.2016.06.017 (2016).

8 Ba, J. & Kingma, D. Adam: a method for stochastic optimization. In *International Conference on Learning Representations (ICLR).* (2015).
